# Does establishing a preoperative nomogram including ultrasonographic findings help predict the likelihood of malignancy in patients with microcalcifications?

**DOI:** 10.1186/s40644-019-0229-1

**Published:** 2019-07-03

**Authors:** Hongli Wang, Jianguo Lai, Jiao Li, Ran Gu, Fengtao Liu, Yue Hu, Jingsi Mei, Xiaofang Jiang, Shiyu Shen, Fengyan Yu, Fengxi Su

**Affiliations:** 1 Guangdong Provincial Key Laboratory of Malignant Tumor Epigenetics and Gene Regulation Sun Yat-sen Memorial Hospital, Sun Yat-sen University, Yanjiang Road. west No.107, YueXiu district, Guangzhou, Guangdong 510120 People’s Republic of China; 2 Breast Tumor Center, Sun Yat-sen Memorial Hospital, Sun Yat-sen University, Yingfeng Road No. 33, HaiZhu district, Guangzhou, Guangdong 510288 People’s Republic of China; 3 Department of Radiology, Sun Yat-sen University Cancer Center, Dongfeng East Road No.651, Yuexiu District, Guangzhou, Guangdong 510060 People’s Republic of China

**Keywords:** Breast, Microcalcification, Ultrasonography, Nomogram, Mammography

## Abstract

**Background:**

Mammography (MG) is highly sensitive for detecting microcalcifications, but has low specificity. This study investigates whether establishing a preoperative nomogram including ultrasonographic findings can help predict the likelihood of malignancy in patients with mammographic microcalcification.

**Methods:**

Between May 2012 and January 2017, 475 patients with suspicious microcalcifications detected on MG underwent ultrasonography (US). The χ^2^ test was used to screen risk factors among the variables. Then, a multivariate logistic regression analysis was performed to identify independent predictors of malignant microcalcifications. A mammographic nomogram (M nomogram) and mammographic-ultrasonographic nomogram (M-U nomogram) were established based on multivariate logistic regression models. The discriminatory ability and clinical utility of both nomograms were compared by the receiver operating characteristics curve and decision curve analysis. The calibration ability was evaluated using a calibration curve.

**Results:**

Among the cases, 68.2% (324/475) were pathologically diagnosed as breast cancer and 31.8% (151/475) were benign lesions. Based on multivariate logistic regression analysis, age, clinical manifestation, morphology and distribution of microcalcifications on MG and lesions associated with microcalcifications on US were confirmed as independent predictors of malignant microcalcifications. In terms of discrimination ability, the C-index of the M-U nomogram was significantly higher than that of the M nomogram (0.917 vs 0.897, *p =* 0.006). The bias-corrected curve was close to the ideal line in the calibration curve. Decision curve analysis suggested that the M-U nomogram was superior to M nomogram.

**Conclusions:**

Combining mammographic parameters with ultrasonographic findings in a nomogram provided better performance than an M nomogram alone, especially for dense breasts, which suggests the value of ultrasonographic finding for individualized prediction of malignancy in patients with microcalcifications.

## Background

Mammography (MG) is highly sensitive for detecting microcalcifications but has low specificity. Only 12.3–42.0% of microcalcifications detected by MG are pathologically proven to be malignant after surgery [1–5]. To appropriately manage patients, the 5th edition of Breast Imaging Reporting and Data System (BI-RADS) suggested subdivisions based on the morphology of suspicious microcalcifications. The guideline indicated that amorphous, coarse heterogeneous, and fine pleomorphic cases should be assessed as category 4B (positive predictive value range: 10–50%), whereas fine linear or fine-linear branching should be assessed as category 4C (positive predictive value range: 50–95%) [6]. However, BI-RADS provides only a range and not exact values of the malignant likelihood of microcalcifications based on morphology. Additionally, unlike morphology, it does not provide recommendations regarding the distribution of suspicious microcalcifications, despite studies showing that the distribution is helpful for predicting malignancy risk [1–5]. Furthermore, combining morphology and distribution descriptors for suspicious microcalcifications has been suggested to provide a more accurate risk stratification [2].

In addition to MG, ultrasonography (US) is one of the most widely available diagnostic options for women with breast tumors [7]. Previous studies have confirmed that malignant microcalcifications are more easily detectable on US than benign microcalcifications [8, 9], and that the visibility of masses on US corresponding to areas of microcalcifications is much higher for highly suspicious microcalcifications [9, 10]. However, no studies have reported differences in the diagnosis of microcalcifications between MG alone and the combination of MG and US. Thus, whether the combined analysis of mammographic features and ultrasonographic findings associated with microcalcifications can better predict the likelihood of malignancy in patients with microcalcifications detected by MG is unknown.

Recently, nomograms which create a simple graphical representation of a predictive statistical model [11], have become widely used as predictive tools for diagnosing malignancies. Timmers et al [12] developed a nomogram for breast cancer based on common mammographic findings on screening mammograms. In addition, Park et al [13] developed a nomogram for predicting underestimation of invasiveness in ductal carcinoma in situ diagnosed by preoperative needle biopsy. These studies suggest that nomograms could be developed for the individualized prediction of malignant microcalcifications. Thus, the purpose of this study was to investigate whether establishing a preoperative nomogram including ultrasonographic findings can help predict the likelihood of malignancy in patients with microcalcifications.

## Methods

### Study population

**Fig. 1:**
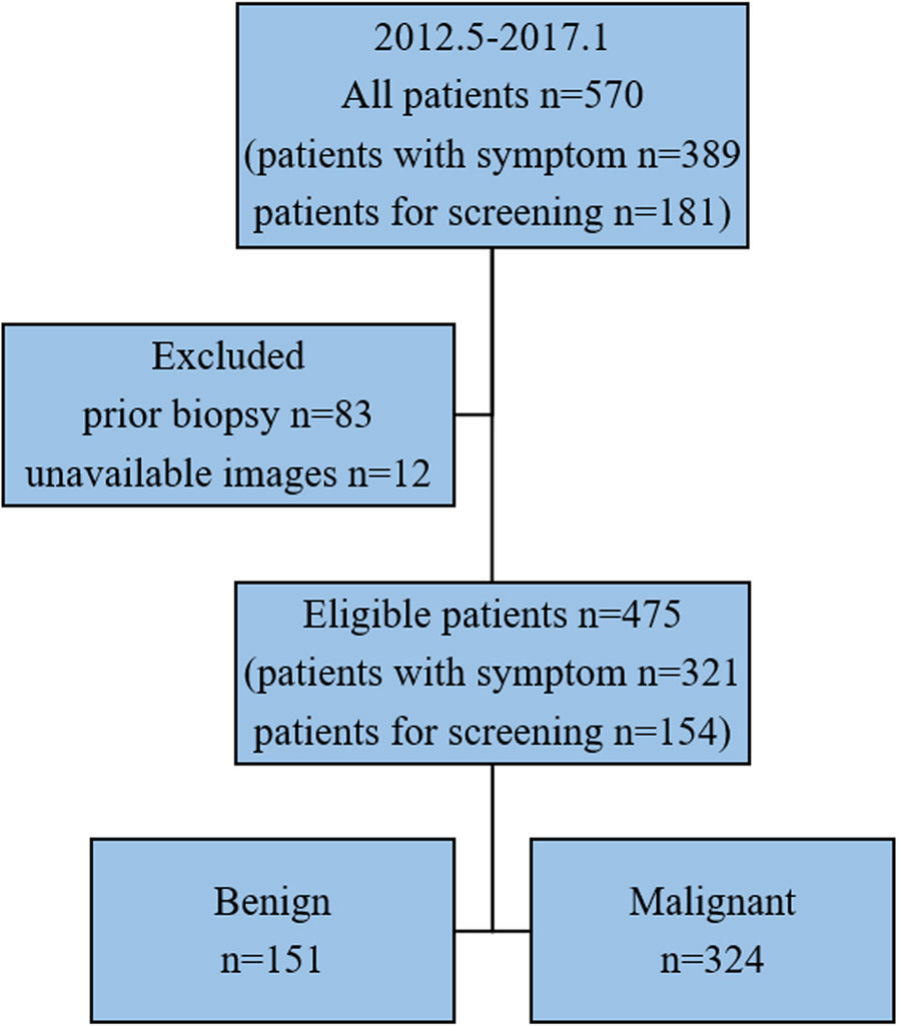
Flow chart of population selection

Our institutional review board approved this retrospective study and waived the requirement for informed consent. We retrospectively reviewed consecutive mammograms taken between May 2012 and January 2017. Suspicious microcalcifications (BI-RADS 4 and 5) without other abnormalities were detected on MG in 570 patients who underwent MG because of symptoms (palpable mass or nipple discharge, 389 patients) or opportunistic screening (181 women). We excluded patients who had undergone prior biopsy (*n* = 83, 62 with symptom and 21 for screening) and patients whose digital images were unavailable for review (*n* = 12, 6 symptomatic and 6 screening). At our institution, breast US is a routine examination for women with breast disease. Thus, a total of 475 lesions from 475 patients (321 with symptom and 154 for screening) were selected from 570 patients in this study (Fig. 1). The median patient age was 45 years (range: 21–79 years).

### Imaging acquisition and analysis

Bilateral digital mammograms with two standard imaging planes (mediolateral oblique and craniocaudal) were obtained using a digital mammographic unit (Planmed Nuance; Planmed, Helsinki, Finland). Breast US examinations were performed with a high-resolution US unit (S2000 or S1000; Siemens Medical Solutions, Erlangen, Germany). All US examinations were performed by one of seven dedicated breast radiologists with a minimum of 3 years of experience. Our radiologists were exclusively devoted to mammographic diagnoses and US scanning and diagnoses, and the US scanning and diagnosis of individual patients were performed by the same radiologist. When suspicious microcalcifications on MG were found before US, the radiologist scanned both whole breasts and focused on the area of microcalcifications. In six patients, when suspicious microcalcifications on MG were found after negative US finding, a “second-look” US scan focused on the area of microcalcifications was performed and microcalcifications were identified in 4 patients. Mammograms and ultrasonograms were simultaneously reviewed by two breast imaging radiologists who were blinded to pathological information but not clinical information. When descriptors differed between two radiologists, consensus was reached after discussion.

Mammograms were reviewed for breast composition and the morphology and distribution of microcalcification according to the BI-RADS Atlas [6]. The morphologies of microcalcifications were divided into two categories: amorphous/coarse heterogeneous or fine pleomorphic/fine linear and fine-linear branching. Additionally, the distributions were divided into two categories: regional/grouped or segmental/linear.

Ultrasonograms corresponding to the microcalcification area on MG and medical records were reviewed for whether the microcalcifications were visible and lesions associated with the microcalcifications which included masses or cysts. A negative finding was recorded when no lesions were found on US.

### Clinical analysis and biopsy

Clinical information, including age and clinical manifestations, was also included in our study. These data were obtained from medical records. Age was divided into three groups: < 40 years, 40–55 years, and > 55 years. Clinical manifestations were classified as palpable mass, nipple discharge, and asymptomatic.

Pathological results, including malignant and benign lesions, were also obtained from medical records. US-guided biopsies (core biopsy with a 14-gauge needle) were performed when microcalcifications were visible on US. Specimen radiography was performed to ensure that the microcalcifications were sampled. Patients with B3 (uncertain malignant potential), B4 (suspicious) or B5 (malignant) biopsy results were referred for open surgery. Patients with benign histopathological findings were referred for vacuum-assisted or surgical excision or follow up. MG-guided wire localization with surgical excision biopsy and specimen radiography were performed when microcalcifications were not found on US. Patients with malignant intraoperative pathological results were referred for breast conservation treatment or modified mastectomy. Patients with benign histopathological findings had a minimum of 12 months follow-up after US- or MG-guided biopsy or surgery. Follow-up data were queried through April 2, 2018.

### Statistical analysis

The χ^2^ test was used to screen risk factors from variables. Then, a multivariate logistic regression analysis was performed to identify the independent predictors of malignant microcalcifications. The mammographic nomogram (M nomogram) and mammographic-ultrasonographic nomogram (M-U nomogram) were established to predict the likelihood of malignancy in patients with microcalcifications given the predictors in the final multivariable model. The discrimination, calibration ability, and clinical usefulness were used to evaluate the model’s performance. The discriminative abilities of both nomograms were quantified by ROC curve and concordance index (C-index) measures. The calibration curve and Hosmer–Leme-show goodness-of-fit test were used to determine the calibration ability of the model. Decision curve analysis (DCA) was executed to assess the clinical utility of both nomograms by quantifying the net benefits for a range of threshold probabilities. *P* < 0.05 was indicative of a statistically significant difference. Stata/MP version 13.0 (StataCorp LP, College Station, TX, USA) and R software version 3.3.2 were used to conduct all statistical analyses.

## Results

### Pathological results

Among the 475 cases (321 symptomatic and 154 screening) selected from 570 patients (389 symptomatic and 181 screening), 324 (68.2%) were malignant, while 151 (31.8%) were benign. Pathological results are shown in Table 1.

**Table 1:** Pathological Results of Microcalcifications Detected by MG

Pathological Results	Patients (n)	Percent (%)
Malignant	324	68.2
IDC	211	44.4
DCIS	111	23.4
ILC	2	0.4
Benign	151	31.8
Fibrocystic change	39	8.2
Adenosis	34	7.2
Ductal hyperplasia without atypia	32	6.7
Atypical ductal hyperplasia	16	3.4
Papilloma	12	2.5
Fibroadenoma	11	2.3
Radial scar/ complex sclerosing lesion	7	1.5

*IDC* invasive ductal carcinoma, *DCIS* ductal carcinoma in situ, *ILC* invasive lobular carcinoma

### Predictors of malignant microcalcifications

Predictors associated with a higher likelihood of malignant microcalcifications in the χ^2^ test included age, clinical manifestation, morphology and distribution of microcalcifications on MG, whether the microcalcifications were visible, and lesions associated with the microcalcifications on US (Table 2). Sequentially, considering the multivariate logistic regression analysis, age, clinical manifestation, morphology and distribution of microcalcifications on MG, and lesions associated with microcalcifications on US were confirmed to be independent predictors of malignant microcalcifications (Table 2).

**Table 2:** The χ^2^ Test and Multivariate Logistic Regression Results for Predicting the Malignant Likelihood of Microcalcifications Detected by MG

Variables	Cancer (*n*=324)	Benign (*n*=151)	χ2 test	Multivariate analysis	
			*P* Value	*P* Value	OR (95%CI)
Age (y)			0.005		
<40	80 (24.7)	43 (28.5)			Reference
40-55	189 (58.3)	99 (65.5)		0.026	2.12 (1.10,4.09)
>55	55 (17.0)	9 (6.0)		0.004	5.17 (1.71, 15.61)
Clinical manifestation			<0.001		
Mass/nipple discharge	285 (88.0)	36 (23.8)			Reference
Asymptomatic	39 (12.0)	115 (76.2)		<0.001	0.11 (0.05, 0.21)
Breast composition			0.237		
a + b	19 (5.9)	5 (3.3)			
c + d	305 (94.1)	146 (96.7)			
Morphology			<0.001		
Amorphous/coarse heterogeneous	112 (34.6)	101 (66.9)			Reference
Fine pleomorphic/fine linear and fine-linearbranching	212 (65.4)	50 (33.1)		<0.001	3.67 (2.05, 6.59)
Distribution			<0.001		
Regional/grouped	244 (75.3)	138 (91.4)			Reference
Segmental/linear	80 (24.7)	13 (8.6)		0.002	4.51 (1.77, 11.50)
Microcalcifications on US^a^			<0.001		
Yes	301 (92.9)	85 (56.3)			Reference
No	23 (7.1)	66 (43.7)		0.766	1.15 (0.46, 2.89)
Lesions on US ^b^			<0.001		
Mass	300 (92.6)	51 (33.8)			Reference
Cyst/normal	24 (7.4)	100 (66.2)		<0.001	0.12 (0.05, 0.30)

Note: Data are numbers of patients, with percentages in parentheses

*OR* odds ratio

^a^ whether microcalcifications were visible on US

^b^ lesions associated with microcalcifications on US

### Development of the nomograms

**Fig. 2:**
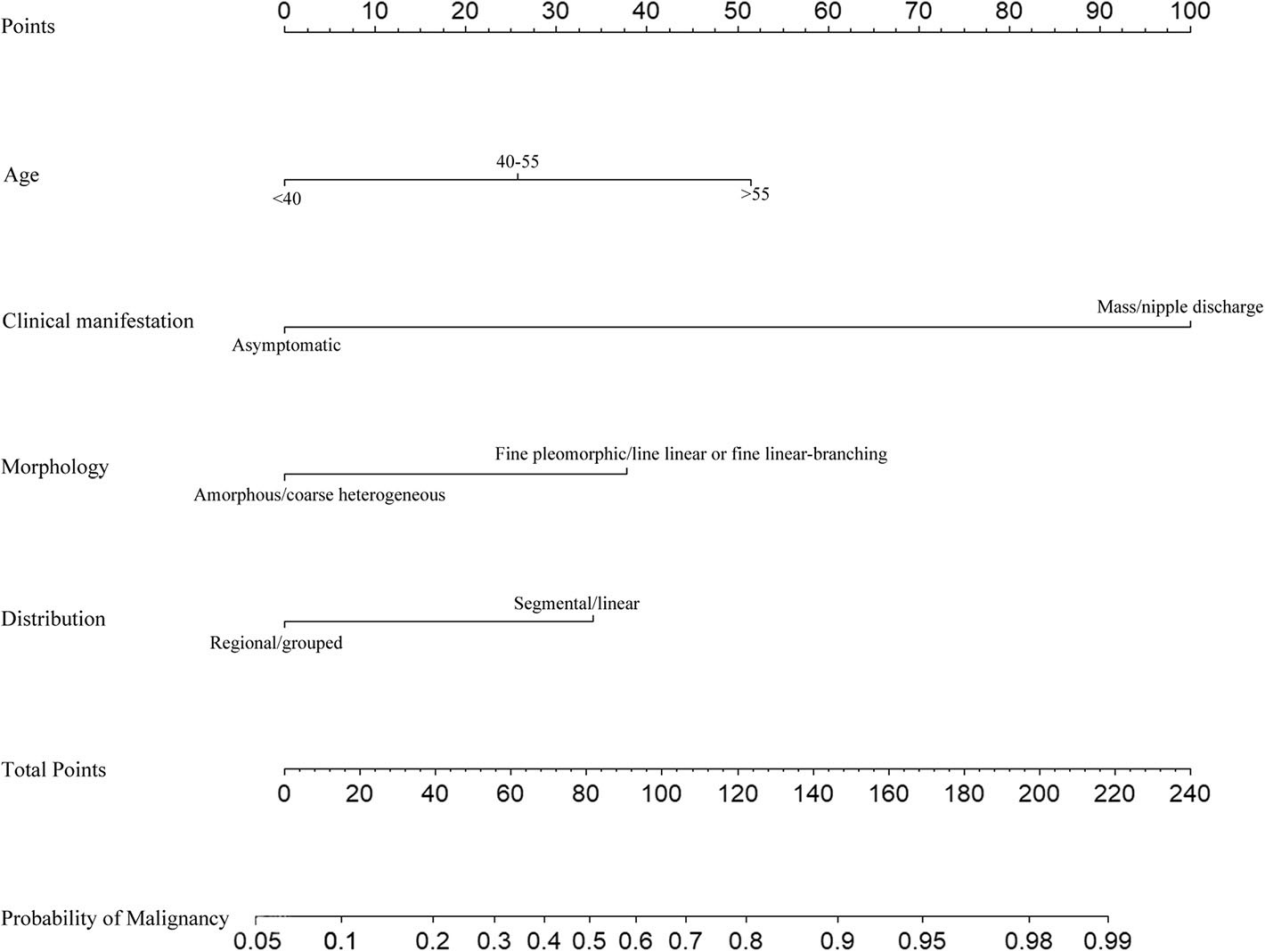
The M nomogram was used to predict the likelihood of malignancy of microcalcifications on MG using age, clinical manifestation, morphology, and distribution of microcalcifications

**Fig. 3:**
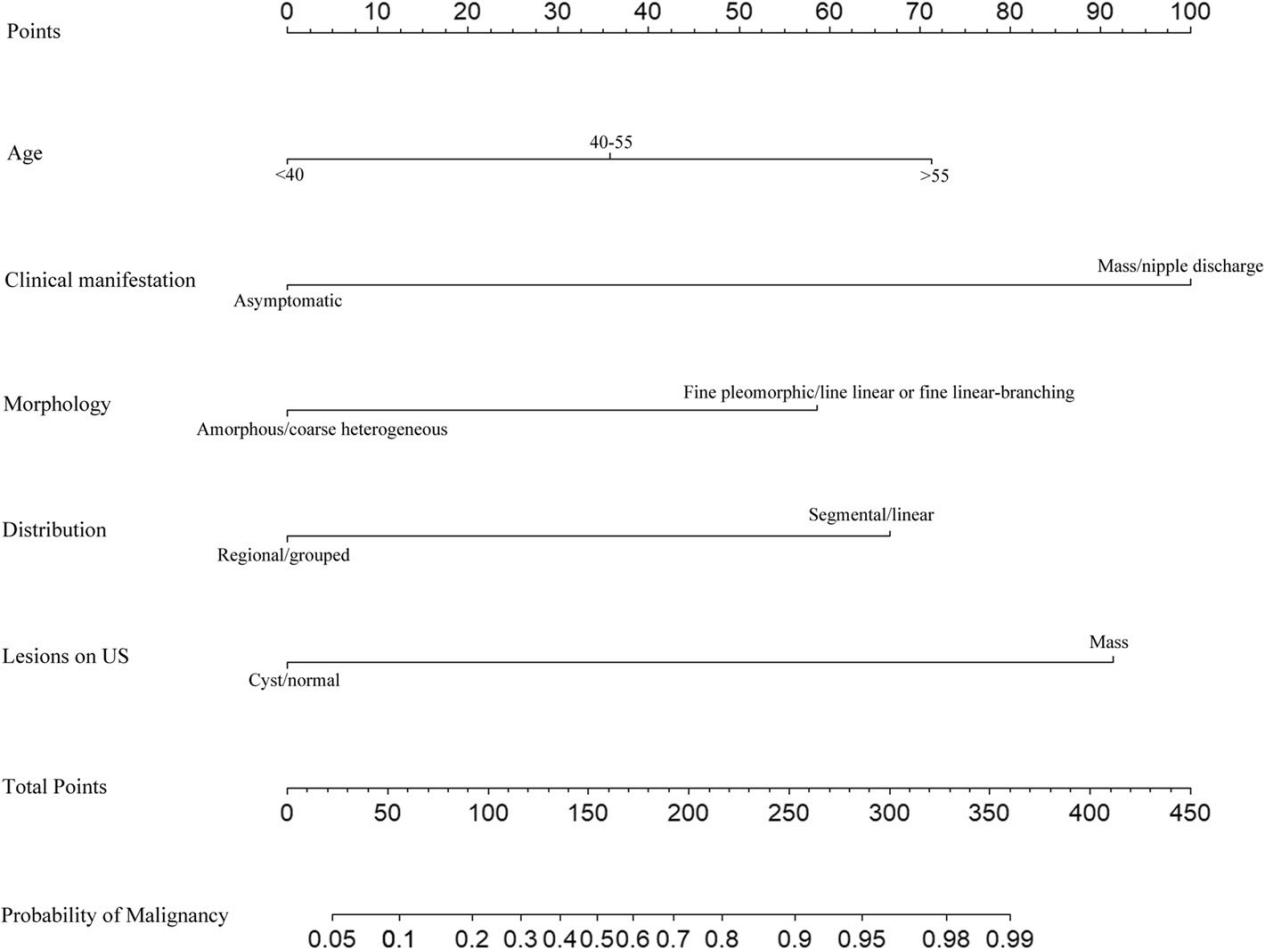
The M-U nomogram was used to predict the likelihood of malignancy of microcalcifications on MG using age, clinical manifestation, morphology and distribution on MG, and lesions associated with microcalcifications on US

Based on the multivariate analysis results, the M nomogram was constructed to predict the likelihood of malignant microcalcifications using four independent predictors (age, clinical manifestation, morphology, and distribution of microcalcifications on MG) (Fig. 2). The M-U nomogram was developed to predict the likelihood of malignant microcalcifications using five independent factors (age, clinical manifestation, morphology and distribution of microcalcifications on MG and lesions associated with the microcalcifications on US) (Fig. 3).

### Performance of the M nomogram and M-U nomogram

**Fig. 4:**
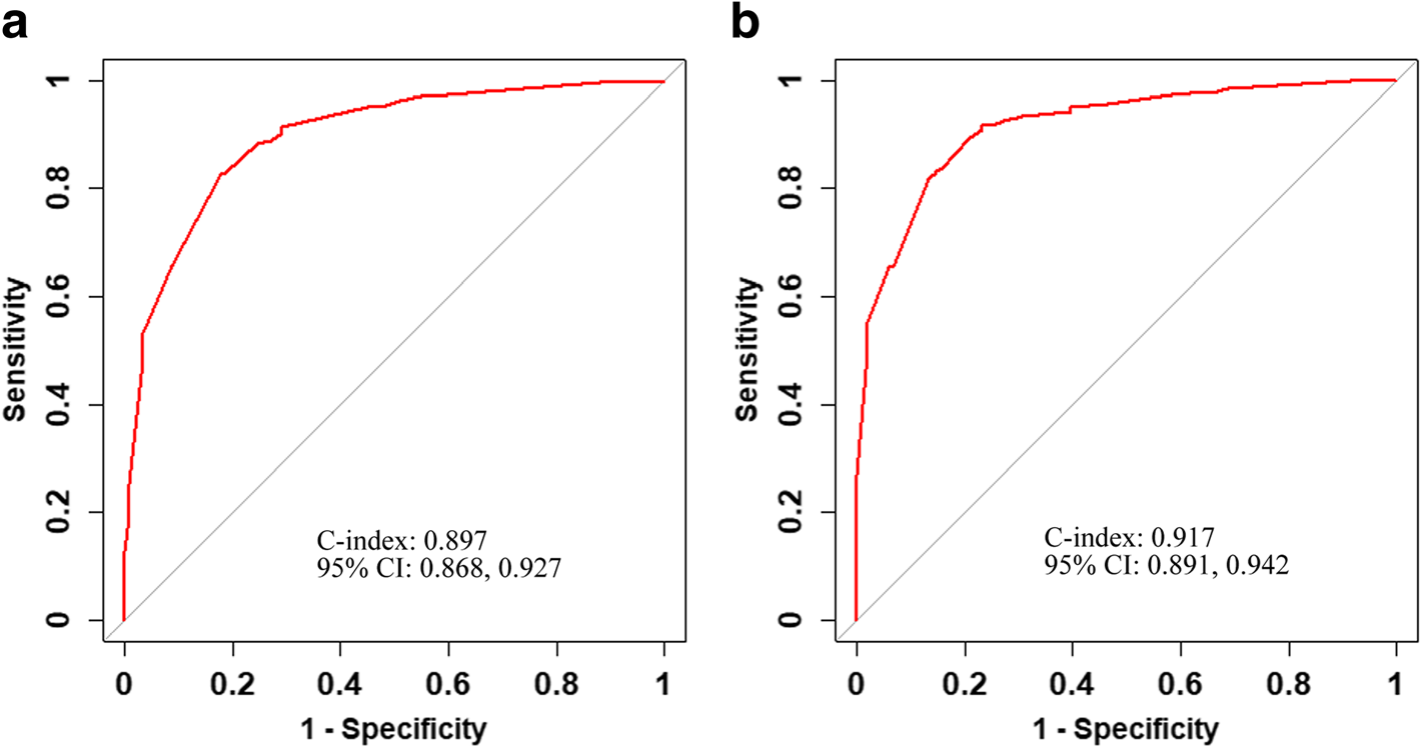
ROC curves and C-index values of the nomogram. **a** The C-index values of the M nomogram were 0.897 (95%CI: 0.868–0.927). **b** The C-index values of the M-U nomogram were 0.917 (0.891–0.942)

**Fig. 5:**
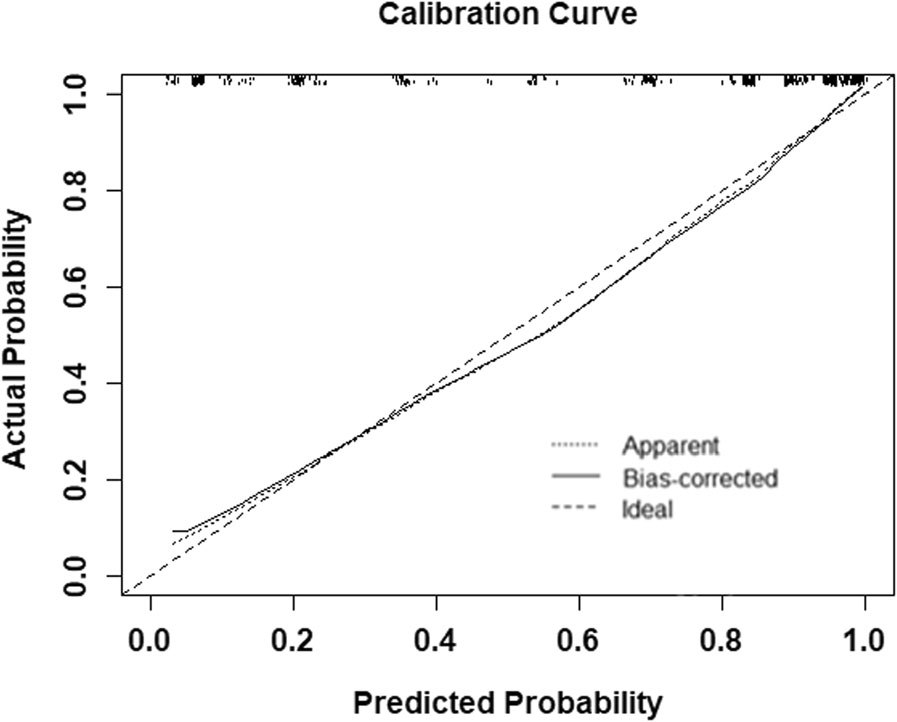
Calibration curve for the M-U nomogram

**Fig. 6:**
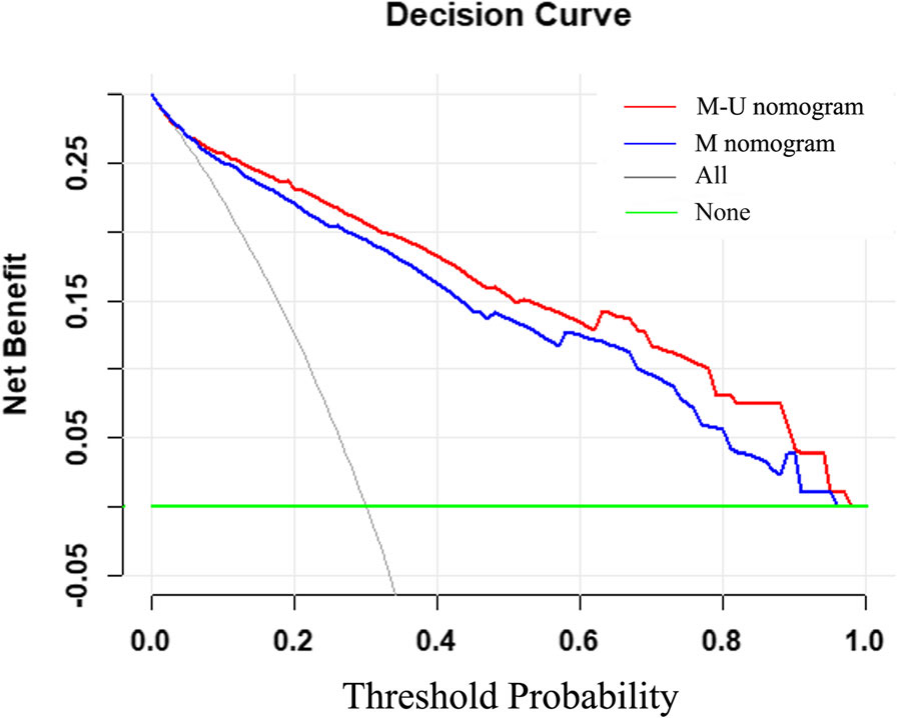
Decision curve analyses for the M nomogram and M-U nomogram. The red line represents the M-U nomogram, and the blue line represents the M nomogram. The gray line represents the hypothesis that all patients had malignant microcalcifications. The green line represents the hypothesis that no patients had malignant microcalcifications. The x-axis represents threshold probability. The y-axis represents net benefit

ROC curves and C-index values were used to test the two nomograms (Fig. 4). The C-index of the M nomogram was 0.897 (95% CI: 0.868–0.927), and the C-index of the M-U nomogram was 0.917 (95% CI: 0.891–0.942). In terms of their discriminatory abilities, the C-index of the M-U nomogram was significantly higher than that of the M nomogram (0.917 vs 0.897, *p =* 0.006). The calibration of the M-U nomogram was performed via a calibration curve (Fig. 5). The bias-corrected curve was close to the ideal line in the calibration curve. The *p*-value of the Hosmer–Leme-show goodness-of-fit test was 0.694. The DCAs of the two nomograms are shown in Fig. 6. With respect to their clinical utility, DCA suggested that the M-U nomogram was superior to the M nomogram across a wider range of threshold probabilities.

## Discussion

In our study, 68.2% of microcalcifications were pathologically diagnosed as breast cancer, and invasive ductal carcinoma was the most common malignant diagnosis (65.1%, 211/324). However, previous studies reported that the positive predictive value of microcalcifications was 12.3–42.0% and that ductal carcinoma in situ was the most common malignant diagnosis [1–5]. The reason for these discrepancies may be differences in the criteria for the inclusion of patients. The previous studies only included asymptomatic patients, whereas our study included asymptomatic patients and patients with a palpable mass or nipple discharge. Park et al [13] reported that a clinically palpable mass suggested invasive cancer.

In this study, we established an M-U nomogram to predict the risk of malignancy in patients with microcalcifications. The C-index of the M-U nomogram was higher than that of the M nomogram (0.917 vs 0.897, *p* = 0.006). Obviously, the discriminatory ability of the M-U nomogram improved when the M nomogram was incorporated along with ultrasonographic findings. The bias-corrected curve was close to the ideal line, which showed that the M-U nomogram was well calibrated. Moreover, DCA demonstrated that the M-U nomogram had good clinical usefulness. Therefore, the M-U nomogram could predict the likelihood of malignancy in patients with microcalcifications with high discrimination, high calibration ability, and robust clinical utility. Finally, we showed that age, clinical manifestation, the morphology and distribution of microcalcifications on MG, and lesions associated with microcalcifications on US were independent predictors of malignant microcalcifications.

In this study, the rate of malignant microcalcifications increased with increasing age (OR = 2.12 in the 40 to 55-year-old cohort and OR = 5.17 in the > 55-year-old cohort). Farshid et al. [14] also reported that older women had a higher rate of malignancy (41.7% in the 40 to 49-year-old cohort, 48.1% in the 50 to 59-year-old cohort, 48.3% in the 60 to 69-year-old cohort, and 56.5% in the > 70-year-old cohort); however, in their study, age was not an independent predictor. The age peak in our study was between 40 and 55 years, which is consistent with a report that the peak age of breast cancer onset is between 45 to 55 years in China [15]. Apart from age, it is also necessary to record clinical manifestation before MG. Especially in China, clinical manifestation was often missing because of the large number of patients. Compared with asymptomatic women, patients with palpable masses or nipple discharge were more likely to show malignant microcalcifications (*P* < 0.001). Farshid et al. [14] reported that when a palpable mass was associated with microcalcifications, the microcalcifications had a higher rate of malignancy. The cause of pathologic nipple discharge is primarily benign lesions, but there is still a possibility of malignancy [16]. Pathologic nipple discharge patients with breast microcalcifications had a higher risk of breast cancer than those without breast calcifications (*p* = 0.000) [17].

On MG, the morphology and distribution of microcalcifications were helpful for predicting malignant risk, as was found in previous studies [1–5, 18]. Moreover, in both previous studies and our study, the morphology and distribution descriptors showed an increasing trend toward malignancy from amorphous/coarse heterogeneous to fine pleomorphic/fine linear/fine-linear branching microcalcifications and from regional/grouped to segmental/linear [1–4]. In addition to asymptomatic patients, our study included patients with a palpable mass or nipple discharge. However, microcalcifications were the only abnormality found on mammogram. For the detection of a palpable mass, breast density is the strongest predictor for mammography failure [19]. Mammographic sensitivity declines significantly with increasing breast density [20–23], from a level of 85.7–88.2% in women with almost entirely fatty breasts to 62.2–68.1% in women with extremely dense breasts [22, 23]. In our study, 94.9% (451/475) of patients had heterogeneously or extremely dense breasts. Dense breast may lead masking of a palpable mass on mammography. For microcalcification biopsy, mammography-guided stereotactic vacuum assisted biopsy is a reliable, safe and accurate method for tissue sampling and evaluation of breast lesion including suspicious microcalcifications [24–26]. However, the stereotactic guided biopsy device was not available in our institute. Consequently, MG-wire guided localization with surgical excision biopsy and specimen radiography were performed.

Compared with MG, US cannot be used alone for diagnosing microcalcifications. However, it is helpful for predicting malignant risk [8–10]. As reported by Moon et al. [10], masses associated with microcalcifications on US were confirmed to be an independent predictor of malignant microcalcifications. Moon reported that US depicted more breast masses associated with malignant than with benign microcalcifications (82%, 31/38 vs 23%, 14 /62, *P* < 0.001), while the proportion of breast masses associated with malignant and benign microcalcifications identified by US in our study was 92.6% (300/324) and 33.8% (52/151) (*P* < 0.001), respectively.

Our study had some limitations. First, a validation group was absent because of the retrospective nature of this small-sample study. However, DCA demonstrated that the M-U nomogram had good clinical usefulness. Second, the extent of microcalcifications was not considered, because, according to BI-RADS, the distribution of microcalcifications considers the extent. For example, 2 cm is the boundary between the regional and the group distribution. Therefore, the extent and distribution of microcalcifications are not independent of each other. Third, we did not perform comparisons with old films, because we included only patients with first mammogram. And, we hoped that the establishment of a nomogram would contribute to the initial diagnosis of breast microcalcifications.

## Conclusions

In conclusion, combining mammographic parameters with ultrasonographic findings in a nomogram provided better performance than an M nomogram alone, especially for dense breast, which suggests the value of ultrasonographic findings for individualized prediction of malignancy in patients with microcalcifications.

## Data Availability

The datasets used and/or analyzed during the current study are available from the corresponding author on reasonable request.

## Abbreviations

BI-RADS: Breast imaging reporting and data system
C-Index: Concordance index
DCA: Decision curve analysis
M nomogram: Mammographic nomogram
MG: Mammography
M-U nomogram: Mammographic-ultrasonographic nomogram
US: Ultrasonography
